# Engineering geminivirus resistance in *Jatropha curcus*

**DOI:** 10.1186/s13068-014-0149-z

**Published:** 2014-10-21

**Authors:** Jian Ye, Jing Qu, Hui-Zhu Mao, Zhi-Gang Ma, Nur Estya Binte Rahman, Chao Bai, Wen Chen, Shu-Ye Jiang, Srinivasan Ramachandran, Nam-Hai Chua

**Affiliations:** Temasek Life Sciences Laboratory, NO.1 Research Link, National University of Singapore, Singapore, 117604 Singapore; State Key Laboratory of Plant Genomics, Institute of Microbiology, Chinese Academy of Sciences, NO.1 Beichen West Road, Beijing, 100101 China; Laboratory of Plant Molecular Biology, Rockefeller University, 1230 York Avenue, New York, NY 10021 USA

**Keywords:** Biodiesel, Indian cassava mosaic virus, *Jatropha curcus*, Virus resistance, Transgenic

## Abstract

**Background:**

*Jatropha curcus* is a good candidate plant for biodiesel production in tropical and subtropical regions. However, *J. curcus* is susceptible to the geminivirus Indian cassava mosaic virus (ICMV), and frequent viral disease outbreaks severely limit productivity. Therefore the development of *J. curcus* to carry on durable virus resistance remains crucial and poses a major biotechnological challenge.

**Results:**

We generated transgenic *J. curcus* plants expressing a hairpin, double-stranded (ds) RNA with sequences homologous to five key genes of ICMV-Dha strain DNA-A, which silences sequence-related viral genes thereby conferring ICMV resistance. Two rounds of virus inoculation were conducted via vacuum infiltration of ICMV-Dha. The durability and heritability of resistance conferred by the dsRNA was further tested to ascertain that T1 progeny transgenic plants were resistant to the ICMV-SG strain, which shared 94.5% nucleotides identity with the ICMV-Dha strain. Quantitative PCR analysis showed that resistant transgenic lines had no detectable virus.

**Conclusions:**

In this study we developed transgenic *J. curcus* plants to include a resistance to prevailing geminiviruses in Asia. These virus-resistant transgenic *J. curcus* plants can be used in various *Jatropha* breeding programs.

**Electronic supplementary material:**

The online version of this article (doi:10.1186/s13068-014-0149-z) contains supplementary material, which is available to authorized users.

## Background

*Jatropha curcas*, a small woody plant belonging to the *Euphorbiaceae* family, is a non-food oil seed crop mainly grown in the tropical and subtropical regions. This plant possesses several traits render this woody plant suitable for biodiesel feedstock production. It is easy to propagate and grows rapidly. It has a short gestation period, low seed cost and high oil content. Moreover, the ability of *J. curcus* to thrive on degraded soil and its wide adaptability to different growth conditions allows the use of marginal or non-arable wasteland for the application of this plant on an industrial scale. However, the productivity of *J. curcus* in the field is limited by the occurrence of *Jatropha curcas* mosaic disease (JcMD) [1-3]. The disease incidence is particularly significant in the Indian subcontinent; about 25% in northern India [1] and up to 47% in southern India [2].

We have previously reported the first full-length genome sequence of one geminivirus, a strain of Indian cassava mosaic virus (ICMV-Dha), as the causative pathogen of JcMD found in Southern India [3]. Following our report, three other related geminiviruses were isolated from *Jatropha* plants in Africa and South Asia [4-6]. Recently, we reported another highly pathogenic ICMV Singapore strain as the causative agent for JcMD in South-east Asia which shares a 94.5% identity with ICMV-Dha [7]. The recurrent identification of ICMV as an epidemic viral pathogen in various *Jatropha* plantations prompted us to investigate the biology of the virus in more detail.

Geminiviruses, which are single-stranded DNA viruses infecting a range of economically important crop species (such as cassava, maize, cotton and tomato) in tropical and subtropical regions, have become a major threat to world agriculture in the past decade [8]. Based on genome organization, insect vector and host range, the family *Geminiviridae* can be classified into four genera: *Begomovirus, Mastrevirus*, *Curtovirus* and *Topocuvirus*. So far, all five *Jatropha* viral pathogens belong to one genera: *Begomovirus*. Most of these viruses contain two genomic components termed DNA-A and DNA-B (approximately 2.7 to 3.0 kb) and they are all exclusively transmitted via the whitefly, *Bemisia tabaci.* The virus DNA-A-positive strand encodes the coat protein (CP/AV1) involved in the encapsidation of viral DNA, virus movement and viral transmission by *B. tabaci*). Among other encoded proteins, the replication associated-protein (Rep) AC1 is absolutely required for the replication of both genomic components. The transcriptional activator protein (TrAP) AC2 is needed for transcriptional activation of viral gene transcription and plant host gene expression. The replication enhancer protein (Ren) AC3 greatly enhances viral DNA accumulation by interacting with Rep/AC1. Another viral protein, AC4, acts as a gene-silencing suppressor to compromise the host defense system. All these five genes are essential for the virus life cycle and pathogenesis [8].

Because of the capacity of geminiviruses to evolve rapidly by mutation, recombination and pseudo-recombination, the development of plants with durable virus resistance continues to be a major challenge. One strategy involves genetic crossing of resistant and susceptible *Jatropha* germplasms. This strategy has the advantage that segregation patterns can be clearly observed between resistant and susceptible lines [9]. However, germplasm-mediated resistance via crossbreeding is time-consuming and requires a large number of progeny plants (large-scale field tests) to ascertain segregation patterns in future generations [9]. Therefore, transgenic technology has been considered as the method of choice for improving the virus resistance of *J. curcus*. Recently, we and other groups have established transformation platforms which facilitate the transfer of foreign genes into the *J. curcus* genome [10-13], and we have used this method to produce virus-resistant transgenic *J. curcus.*

A major strategy to produce transgenic plants with virus resistance is based on the concept of pathogen-derived resistance (PDR) in which the transgene is derived from viral sequences. The mechanism of PDR includes protein-mediated resistance and RNA interference (RNAi). Both mechanisms have been shown to confer geminivirus resistance in transgenic tomato, common bean, cassava and others [14-18]. Here, we report the production of several JcMD-resistant *J. curcus* lines by expressing a hairpin double-stranded (ds) RNA targeting five key geminivirus DNA-A genes. Some of the transgenic lines displayed broad resistance to related geminiviruses, with 94.5% nucleotide identity in the transgene sequences.

## Results

### Hairpin, double-stranded RNA construct

**Figure 1:**
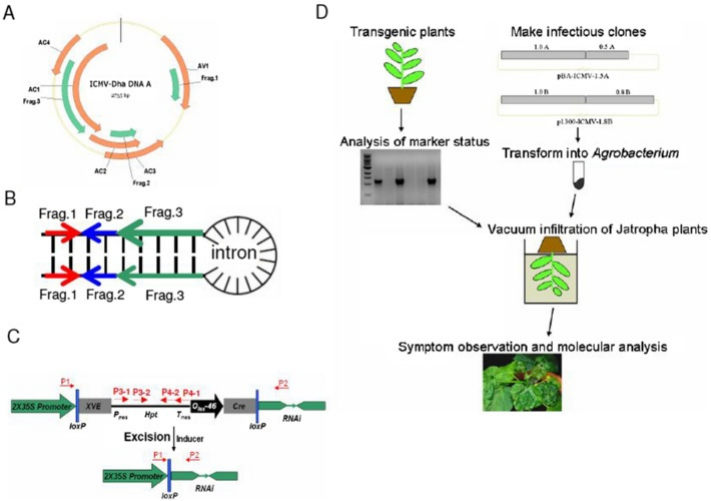
**Schematic diagrams of the transformation vector and experimental procedure. (A)** Selection of three DNA fragments targeting different viral genomic regions. **(B)** The three fragments were ligated and assembled into a sequence which, when transcribed, would produce a hairpin RNAi structure. Arrows indicate the orientation of each fragment in the hairpin structure. **(C)** Schematic diagram of the structural features of the inducible pX9- hpICMV RNAi construct and *Cre*/*loxP*-mediated DNA recombination (Zuo *et al*. Guo *et al*. [19,20]). Cre, the bacteriophage P1 Cre recombinase with an intron; Frag, fragment; *Hpt*, a hygromycin-resistance marker gene driven by nopaline synthase (nos) promoter (Pnos); ICMV, Indian cassava mosaic virus; *loxP*, specific recognition sites of *Cre*; *OlexA-46*, eight copies of the *LexA* DNA-binding site fused to the −46 CaMV 35S promoter; XVE, a chimeric transactivator containing the regulator domain of an estrogen receptor. Arrows inside transcription units indicate the direction of transcription. P1 to P4 denote primers used for PCR analysis to a detect recombination event for *Hpt* selection marker excision shown in Figure 2. **(D)** A flowchart of experimental procedure for screening virus resistance transgenic events by *Agrobacterium*-mediated infection.

We have previously identified the causal pathogen for the JcMD in Southern India as the strain of ICMV known as ICMV-Dha [3]. We chose the sequences of this ICMV-Dha strain to engineer virus resistance via RNAi technology. Three viral gene fragments were ligated to generate the sense and antisense arms in the hairpin dsRNA. Fragment 1 (250 bp) targets the gene encoding CP/AV1 and the *AC5* gene, fragment 2 (250 bp) targets genes for TrAP/AC2 and Ren/AC3 and fragment 3 (609 bp) targets genes for Rep/AC1 and AC4 (Figure 1A). The ligated fragment (fragment 1, 2 and 3) with the designated orientation as indicated by an arrow has the potential to generate a hairpin dsRNA structure with an intron (Figure 1B). The siRNA pool, produced from the hairpin (hp) RNA, should have the potential to silence five key viral genes encoding AC1 (Rep, Rep Replication associated protein), AC2 (TrAP, transcriptional activator protein), AC3 (Ren, (Replication enhancer protein), AV1 (Coat protein, CP) and AC4. We placed this hpRNA-encoding DNA fragment into a chemical-inducible marker excision vector which has been shown to function in *J. curcus* [13].

We then replaced the original G10-90 promoter with a Cauliflower mosaic virus (CaMV) 35S promoter with double enhancers and named it pX9-hpICMV RNAi. Upon chemical induction with 17β-estradiol, *Cre/lox P* mediated recombination excised the DNA fragment containing the hygromycin-resistance gene. This recombination event resulted in placing the hpRNA-encoding DNA fragment immediately downstream of the 35S promoter [13]. The PCR product using primer 1 and 2 should be approximately 6 kb before chemical induction and approximately 1.2 kb after induction. However, the approximately 6 kb product could not be amplified using our PCR program. The absence of PCR products using primer 3 and 4 indicated the excision of the hygromycin phosphotransferase (*Hpt*) gene (Figure 1C).

### Plant transformation and virus inoculation

**Figure 2:**
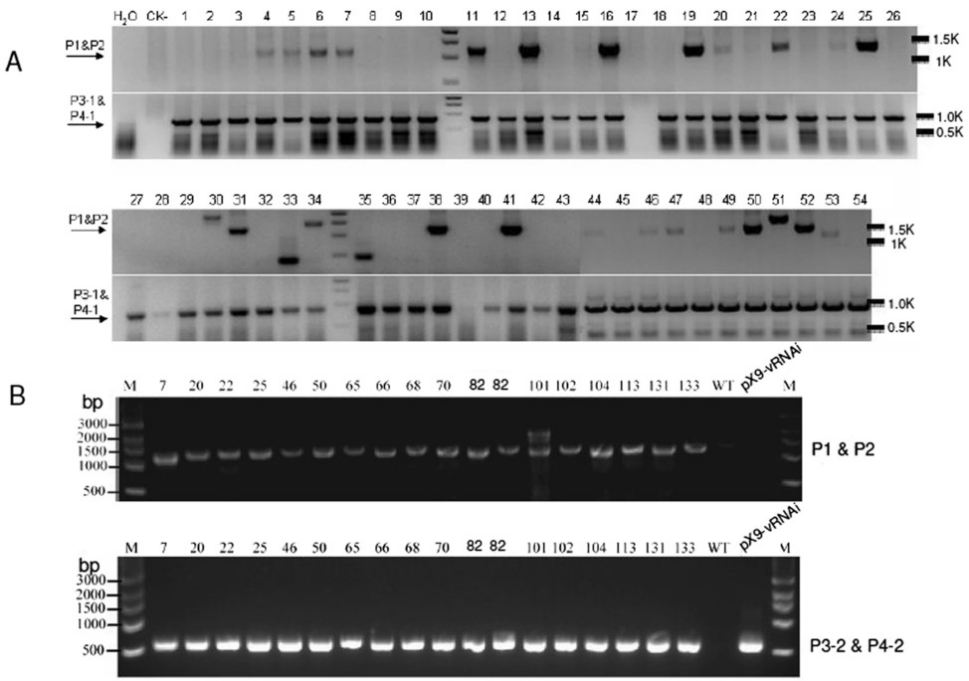
**PCR analysis of genomic DNA of wild-type (WT) and transgenic plants. (A)** Upper panel: the expected PCR products using P1 and P2 primers indicating the occurrence of marker excision events. H2O control, no plant genomic DNA was added. CK- , non-transformed WT control plants. Lower panel: PCR products using P3 and P4 primers indicating the presence of the hygromycin resistance gene. Number indicates transgenic line number. **(B)** WT and resistant transgenic lines after the first virus challenge. M: DNA size marker (bp), pX9-vRNAi: pX9-hpICMV RNAi plasmid. Numbers refer to independent (17) transgenic lines.

The pX9-hpICMV RNAi vector was transformed into *Agrobacterium* and a total of 133 T0 transgenic plants were generated. We performed PCR analysis on genomic DNA using two pairs of primers (P1-P2 and P3-1 together with P4-1, Figure 1C). A total of 53 out of 133 plants showed a 1.2 kb product using primers P1 and P2, indicating the occurrence of successful marker excision. However, the presence of PCR products of P3-1 and P4-1 corresponding to the *HPT* gene suggested that all 53 transgenic plants were chimeric. Figure 2A shows the results of transgenic lines 1 to 54. We purified the PCR products from the P1-P2 primer of line 13, 16, 19, 25, 31, 38, 50, 52 and cloned into T-vector. DNA sequencing of these products confirmed the positive bands were true marker-free products (data not shown). Transgenic plants which were positive for P1-P2 products were used for virus challenge by vacuum infiltration with *Agrobacterium*-mediated virus infection (Figure 1D).

**Figure 3:**
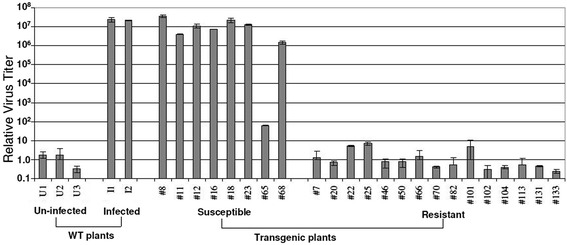
**Quantitative PCR analysis of virus titer in WT and transgenic plants after the first virus challenge.** Plants were inoculated with virus by vacuum infiltration. Uninfected WT plants were used as controls and the average control value was set as 1. I, infected WT plants; U, uninfected WT plants; WT, wild-type.

**Figure 4:**
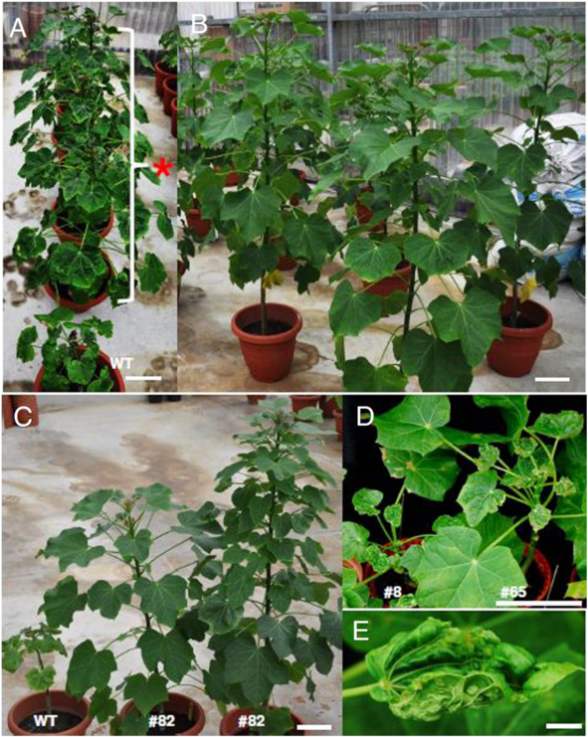
**Symptoms of plants after the first virus challenge**. Bar: 10 cm except 1 cm in **(E). (A)** WT and susceptible transgenic lines (indicated as *) with virus symptoms. **(B)** Putative resistant transgenic lines. **(C)** Comparison of infected WT and resistant transgenic lines. Left: infected WT plant with virus symptoms; middle and right: two T0 transgenic plants from the same transgenic event line 82 with virus resistance. **(D)** Two different lines with different severity of virus disease symptoms; severe line 8 and mild line 65. **(E)** Severe symptoms on leaf of line 8.

Two and a half months after the ICMV-Dha virus challenge, inoculated WT and susceptible transgenic plants showed very obvious curling and a mosaic phenotype on systemic leaves. We extracted genomic DNA and performed quantitative PCR to investigate virus titers in transgenic and WT plants. Uninfected WT plants were used as controls, and the average value of these control plants was set as 1. There was no cycle threshold (Ct) value difference between symptomless resistant transgenic plants and uninfected control plants. In total, 15 independent transgenic lines presented with the symptomless phenotype and no virus could be detected in these resistant transgenic plants. However, we detected very high virus titers in susceptible transgenic plants, which were similar to those found in infected WT plants (Figure 3). Six months after virus inoculation, the susceptible transgenic plants became obviously stunted with malformations and plant size reduction, whereas the resistant transgenic plants grew normally (Figure 4). The virus titers correlated very well with the severity of virus symptoms. A much lower virus amount was detected in plants with mild symptoms (for example line 65) compared with plants with strong symptoms (for example line 8) (Figure 3 and Figure 4D, E). We collected samples from the resistant T0 plants and performed PCR analysis to confirm the occurrence of a marker excision event (Figure 2B).

**Figure 5:**
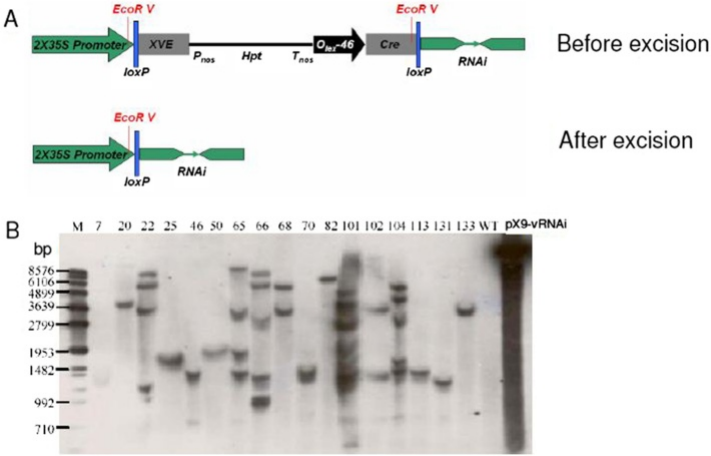
**Analysis of transgene copy number by Southern blotting. (A)** Schematic diagram of marker-free vector-based hairpin RNAi structure. **(B)** Southern blot using a probe carrying the CaMV 35S double enhancers. WT control or transgenic plant DNAs were digested with *Eco*RV. Probe prepared from the 35S double enhancers detected the transgene copy number irrespective of the marker status. Cre, the bacteriophage P1 Cre recombinase with an intron; Frag, fragment; *Hpt*, a hygromycin-resistance marker gene driven by nopaline synthase (nos) promoter (Pnos); ICMV, Indian cassava mosaic virus; *loxP*, specific recognition sites of *Cre*; *OlexA-46*, eight copies of the *LexA* DNA-binding site fused to the −46 CaMV 35S promoter; XVE, a chimeric transactivator containing the regulator domain of an estrogen receptor. Arrows inside transcription units indicate the direction of transcription.

Next, we performed Southern blotting to examine the transgene copy number in resistant lines. As there are two EcoRV sites around the two loxP sites, the genomic DNAs were digested with EcoRV (Figure 5A). Southern blots using probe prepared from the 35S promoter detected the copy number of the transgene irrespective of the marker status. Figure 5B shows that resistant T0 plant lines 7, 20, 25, 50, 82, 113, 131 and 133 contained a single copy of the transgene. Along with quantitative PCR (qPCR) analysis of viral titers and symptomatic observations, we considered these transgenic plants to be candidate-positive resistant plants.

### Heritable virus resistance in T1 plants

**Figure 6:**
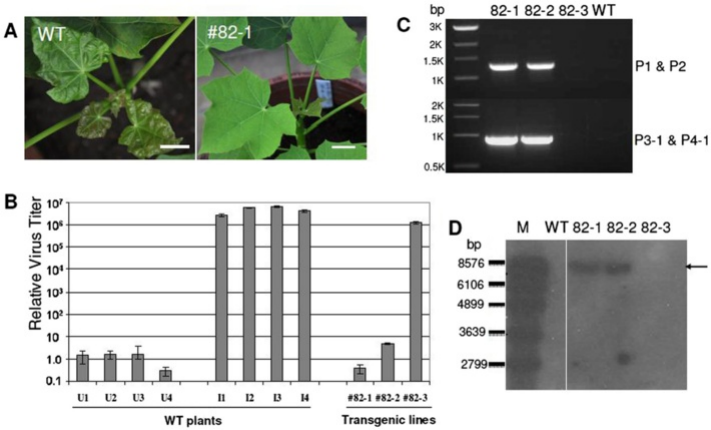
**Heritable virus resistance trait in T1 transgenic plants. (A)** Virus symptoms in WT control plants and progeny plants of transgenic line 82–1 after infection of ICMV-Dha . Bar: 1 cm. **(B)** Quantitative PCR analysis of virus titers after virus challenge. Uninfected (U1 to U4) WT plants were used as controls and the average control value was set as 1. Inoculated (I1 to I4) WT plants were used as positive controls. **(C)** PCR analysis of genomic DNAs prepared from WT and transgenic lines. Upper panel: PCR product using P1 and P2 primers. Lower panel: PCR product using P3-1 and P4-1 primers. **(D)** Southern blotting using a probe carrying the CaMV 35S double enhancers for transgenic T1 progeny plants derived from selfing of T0 plant 81. Note that line 82–3 was a null segregant.M: DNA size markers in bp.

We chose number 82 as the first candidate for further characterization as two line 82 T0 plants (regenerated from a single transgenic event and confirmed by thermal asymmetric interlaced (TAIL)-PCR sequencing) showed a consistent virus resistance trait (Figure 4C). T1 seeds derived from T0 plant line 82 were germinated along with the WT control. *J. curcus* plants with between three and four true leaves were inoculated with ICMV-Dha. Two months after the virus challenge, WT plants and transgenic line 82–3 showed very obvious virus disease symptoms, whereas transgenic plants 82–1 and 82–2 grew normally (like uninfected control plants) (Figure 6A). qPCR analysis showed the absence of detectable virus in transgenic plants 82–1 and 82–2, but high virus titers were obtained in line 82–3 and WT plants (Figure 6B). To analyze the unexpected virus-sensitive phenotype in line 82–3 we performed PCR analysis and Southern blotting using genomic DNAs isolated from leaf samples taken before the virus challenge. We found that no band was amplified from both P1-P2 and P3-P4, indicating line 82–3 was likely a null segregant (Figure 6C). Southern blotting showed the same bands as T0 transgenic plants in lines 82–1 and 82–2, but no signal was detected for 82–3, confirming it was indeed a null segregant (Figure 6D). Overall, our results show that transgenic plants based on the multi-target dsRNA approach can confer heritable resistance.

### Resistance to a second Indian cassava mosaic virus strain sharing 94.5% nucleotide identity with ICMV-Dha

**Figure 7:**
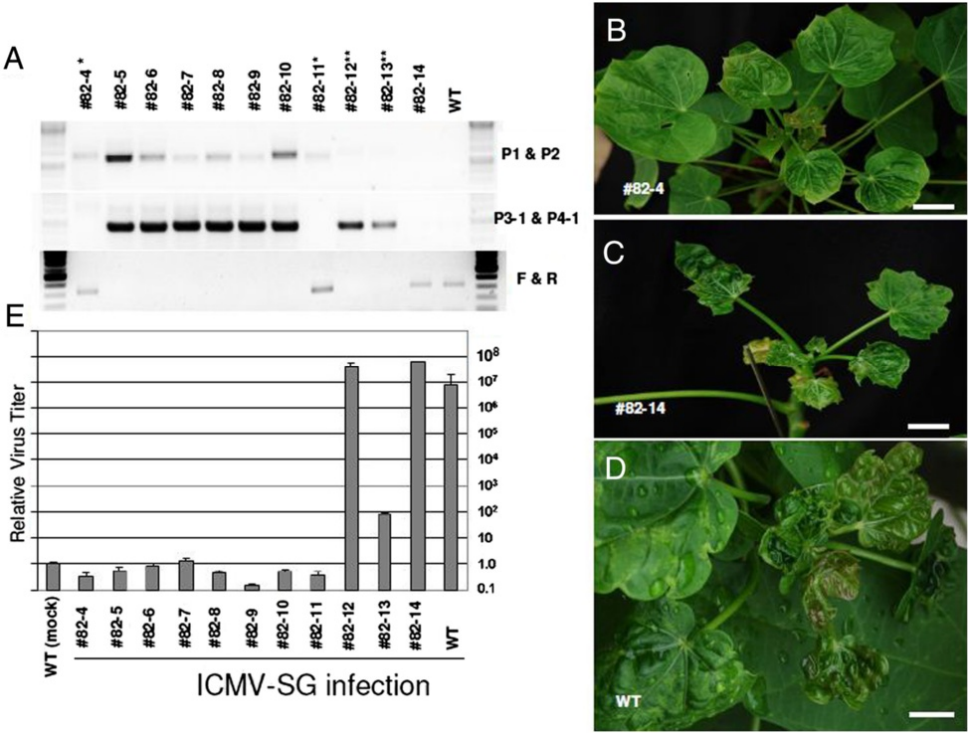
**Virus resistance to infection by ICMV-SG. (A)** PCR analysis of genomic DNAs prepared from WT and transgenic lines. Upper panel: PCR product using P1 and P2 primers. Middle panel: PCR product using P3-1 and P4-1 primers. Lower panel: PCR product using F and R primers which are upstream and downstream flanking sequences, respectively, from the T-DNA (Transferred DNA) insertion position for line 82. Note that lines 82–4 and 82–11 were hemizygous and marker-free, and lines 82–5 to 82–10 were hemizygous but chimeric with respect to the marker gene. Lines 82–12 and 82–13 were homozygous non-marker free, and line 82–14 was a null segregant. **(B)** Virus symptoms in putative homozygous line 82–4, and **(C)** and **(D)**: null segregant plant 82–14 **(C)** and WT JcMD plant **(D)** after vacuum infiltration of ICMV-SG infectious clones. Bar: 10 cm. **(E)** Quantitative PCR analysis of virus titers after virus challenge. WT plants with mock treatment was used as controls and the average control value was set as 1.

We also tested whether the ICMV resistance conferred by hpRNAi has broad virus resistance. We recently reported a second ICMV isolate from the Singaporean *Jatropha* tree (named ICMV-SG, accession number [JX518289]), which shares 94.5% nt homology with the ICMV-Dha strain [7]. Before the virus challenge, we carried out PCR-based genotyping with primer pairs P1-P2 and P3-1, together with P4-1. We found a band of the expected size amplified from P1-P2, but not P3-1 and P4-1, from both lines 82–4 and 82–11, indicating these two plants (82–4 and 82–11) might be marker-free plants, whereas line 82–14 was likely a null segregant as was line 82–3 (Figure 7A). We tested plants of lines 82–4 and 82–11, along with six other plants (lines 82–5 to 82–10), which were still chimeric with respect to the marker gene, for possible resistance to the second virus (ICMV-SG) using the agroinfection-based infection method. As expected, all of the seven plants in which marker excision had occurred were found to be resistant to ICMV-SG in the T1 generation. Further genotype analysis showed that all seven of these plants were hemizygous (lower panel of Figure 7A). The null segregant line 82–14 and two other plants which still retained the marker gene showed typical virus infection symptoms and accumulated high levels of virus genomic DNA, similar to WT control plants (Figure 7B,C,D,E).

**Figure 8:**
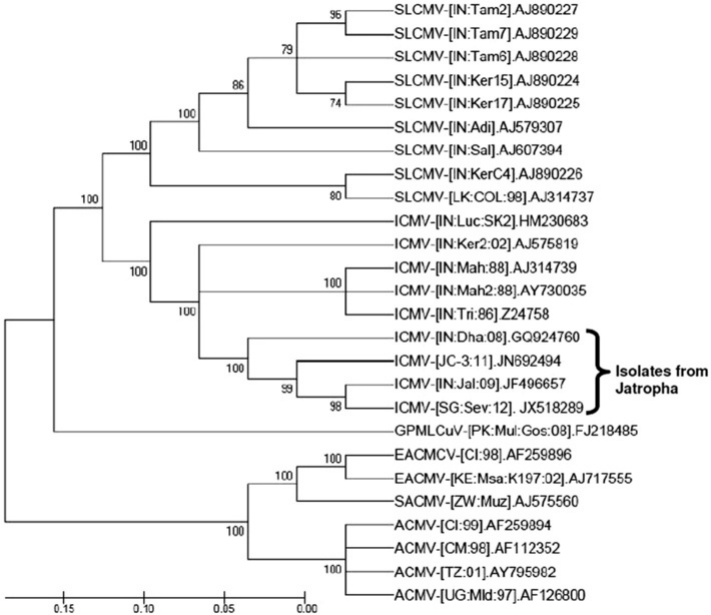
**Phylogenetic relationship of viral isolates recovered from infected** ***J. curcus*** **plants.** Four *J. curcus*-isolates formed one clade in a diagram showing the phylogenic relationship among various geminiviruses. Transgenic lines generated from this report are supposed to be resistant to geminiviruses from this clade. ACMV, African cassava mosaic virus; EACMCV, East African cassava mosaic Cameroon virus; EACMV, East African cassava mosaic virus; SACMV, South African cassava mosaic virus; ICMV, Indian cassava mosaic virus; SLCMV, Sri Lankan cassava mosaic virus; GPMLCuV, Gossypium punctatum mild leaf curl virus. The database accession number of each sequence is given.

We reasoned these ICMV-resistant plants should be also resistant to multiple ICMV strains within a similar evolution clade. Multiple sequence alignment of full-length DNA-A was carried out using the ClustalV program Vector NTI® (Life technologies, Carlsbad, United States) with default parameters. Phylogenetic trees were constructed from multiple alignments using the neighbor-joining method in the MEGA4 program [21], and a bootstrap analysis with 1,000 replicates was performed. Only values above 70 were reported on the trees (Figure 8). Based on the evolutionary phylogenetic tree we deduced that the resistant lines should also contain resistance to another two viral isolates recovered from *J. curcus*.

## Discussion

During the last decade geminiviruses have emerged as one of the major causative pathogens of economically important crops such as cassava, cotton and tomato. As resistance genes are not always available in the relevant crop germplasm, biotechnological approaches have been used to generate transgenic plants to confer virus resistance. Several different strategies have been reported to confer virus resistance to transgenic model plants by expression of: (1) trunked viral proteins, (2) artificial zinc finger nuclease, (3) peptide aptamers capable of binding to viral proteins, (4) non-pathogen-derived antiviral agents and so on [18,22-25]. However, successful applications of these strategies to generate virus-resistant crop plants are rare. In the case of RNA viruses, expression of virus-derived hpRNA has been shown to provide resistance in transgenic plants; more recently, this strategy has been shown to work for DNA viruses such as geminiviruses as well [26]. Successes have been reported with RNAi technology using either single or multiple viral gene sequences, or by artificial miRNA [27-29]. Single target gene approaches have first been used. For example, sequences targeting the *AC1* gene (which encodes the multifunctional Rep protein), the coat protein gene and *AC4* gene have been shown to confer geminivirus resistance in tomato, bean and tobacco plants [14-16,30-32]. In the case of the *Chickpea chlorotic dwarf Pakistan virus* (CpCDPKV), a member of *Mastrevirus* (also a member of the Geminiviridae family) a multi-target approach was adopted. The RNAi trigger sequences include those encoding the N-terminus of the *Rep* gene, the large intergenic (non-coding region; LIR) and the N-terminus of the movement protein gene [32]. Notably, the first field proven transgenic geminivirus-resistant plant-common bean (*Phaseolus vulgaris*) showed that the begomovirus Bean golden mosaic virus (BGMV) can also be suppressed by the expression of a hpRNA transgene derived from *AC1* [33].

Here, we employed an RNAi strategy using hairpin dsRNA to silence five key viral genes of the ICMV. Five viral genes were chosen as targets: genes coding for AC1, AC2, AC3, AV1and AC4. This multi-target strategy conferred transgenic *J. curcus* plants with high resistance to multiple virus infection. After two rounds of virus challenge, resistant plants were free of virus symptoms and no virus could be detected (Figures 4 and 6). Using qPCR to measure and quantify viral titers, we found strong virus resistance in the resistant transgenic lines as compared to WT and susceptible lines (Figures 3, 6 and 7). Our results, together with those of others [30-33], confirm that the hairpin dsRNA-mediated RNAi approach is indeed a promising technology in generating virus-resistant plants and/or conferring stable and effective resistance in plant crops.

An interesting feature of the work presented here is that we were able to challenge the transgenic plants with two distinct viruses (94.5% nt identity for the whole DNA-A), thereby allowing us to ascertain resistance to related viruses. There is 95.3% nt identity between the ICMV-Dha and ICMV-SG strains if we include sequences that we used for the hpRNAi construction. Two other *J. curcus*-isolated ICMV strains, ICMV-JC3 and ICMV-Jalgon, share at least 95% nt identity with the Dha strain sequences that we used for hpRNAi construction (Figure 8 and Additional file 1). In view of this high sequence homology we expect that the transgenic lines generated in this study may also be resistant to these two additional strains. Further field trials are necessary to ascertain whether the multi-target hpRNAi approach can indeed confer durable and heritable resistance to geminiviruses. Nevertheless, here we provide evidences that the multi-target hpRNAi strategy can confer resistance to the ICMV. There were several transgenic T0 lines showing virus resistance to the first ICMV-Dha infection, especially for the line 82. A total of 10 T1 progeny transgenic plants derived from line 82 were free of virus symptoms with no detectable virus, whereas the null segregant 82–3 was as sensitive as WT plants, when challenged with either ICMV-Dha or ICMV-SG strains (Figures 6 and 7). Lines 82–4 and 82–11 are good candidate plants for further commercial development since they are marker-free and contain only a single T-DNA insert. Taken together, our results support and reinforce the notion that the transgenic resistance trait is not only durable but also heritable.

## Conclusions

We generated transgenic *J. curcus* plants with resistance to ICMV via expression of a hairpin dsRNA with sequences homologous to five key ICMV DNA-A genes. Virus resistance was confirmed through two rounds of inoculation with *J. curcus*-isolated strains of ICMV, ICMV-Dha and ICMV-SG. The T1 progeny transgenic plants were virus resistant, confirming that the resistance phenotype was heritable. Transgenic plants generated from this study can be used in various *Jatropha* breeding programs.

## Materials and methods

### Plasmids construction

We replaced the G10-90 promoter in pX7-GFP [20] with a synthetic 35S promoter harboring a double enhancer (detailed sequence in Additional file 2). The resulting vector was named as pX9-GFP. Three fragments of ICMV-Dha DNA-A (Genbank accession number [GQ924760]) were chosen as the target region. The ligated DNA fragment (fragment 1: 521–770 nt, fragment 2: 1210–1459 nt and fragment 3: 1671–2279 nt; see Figure 1B) was generated by two rounds of overlapping extension PCR using the following primers:

- F1-5: 5’-AGCGCCTCGAGCGTTTGAATCTAGACACGATGTGCTC-3’,
- F1-3: 5’-TGCAGTGATGAGTTCCCCTGTGCGTGAACATGTTAAACACCTCACCGAAATCCT-3’,
- F2-5: 5’-GATTTCGGTGAGGTGTTTAACATGTTCACGCACAGGGGAACTCATCACTGCAGC-3’,
- F2-3: 5’-GGAATGTTCCCCATTCCAAGGTATCGAATACCCTCAAGAAACGCCAGGTCTGAG-3’,
- F3-5: 5’-CAGACCTGGCGTTTCTTGAGGGTATTCGATACCTTGGAATGGGGAACATTCCAG-3’,
- F3-3: 5’-AGCGCAAGCTTTAGCTGGAATTGGGCCCTGGATTGCAGA-3’.

Sequences for fragments 1, 2 and 3 were compared with those from related ICMV strains isolated from *J. curcus* (Additional file 1).

The PCR fragment was inserted in the sense orientation into the *Xho*I/*Hin*dIII sites of a pSKint vector [20] to generate pSK-int-sense ICMV. Another fragment, amplified with forward primer 5’-AGCGCGAATTCTAGCTGGAATTGGGCCCTGGATTGCAGA-3’ and reverse primer 5’-AGCGCACTAGTCGTTTGAATCTAGACACGATGTGCTCCA-3’, was subsequently placed in the antisense orientation into the *Eco*RI/*Spe*I sites of pSK-int-sense ICMV to give pSK-int-ICMV RNAi. Finally, the entire RNAi cassette comprising the sense and antisense fragments joined by the *Arabidopsis thaliana Actin* II intron was excised from pSK-int using the flanking *Xho*I/*Spe*I sites and inserted into the *Xho*I/*Spe*I site of pX9-GFP, yielding the construct pX9-hpICMV RNAi (pX9-vRNAi).

### Explant material for transformation

Seeds were obtained from *Jatropha curcas* (JcMD) elite plants (JOil, Singapore) [34]. Cotyledons were harvested from seedlings between five and seven-days-old, cut into small pieces (5 × 5 mm) and used as explants for transformation [13].

### PCR analysis of genomic DNA

Genomic DNAs were prepared with DNeasy plant mini kits (Qiagen, Hilden, Germany) according to the manufacturer’s instructions. Approximately 50 ng of genomic DNA were used for PCR. The reactions were subjected to 94 C for 30 seconds, 60°C for 30 seconds, and 72°C for 2 minutes for 40 cycles. Primers P1 (5’-ATCTCCACTGACGTAAGGGATGAC-3’) and P2 (5’-GTTTAAGATCTACTTACGTAATCAAGC-3’) were used to check the occurrence of marker excision events. DNA fragments containing the hygromycin resistance gene were amplified by either P3-1 (5’-GAGGGCGAAGAATCTCGTGCTTTC-3’) and P4-1 (5’-TACTTCTACACAGCCATCGGTCCA-3’) or P3-2 (5’-GAAGAATCTCGTGCTTTCAG-3’) and P4-2 (5’-CAACCAAGCTCTGATAGAGT-3’). The product amplified by primers P3-1 and P4-1 was 885 bp, whereas the product amplified by primers P3-2 and P4-2 was 745 bp (Figure 1C).

### Virus challenge assay

Infectious clones of ICMV-Dha and ICMV-SG carrying tandem repeats of DNA-A and DNA-B were described [3,7]. Virus challenge assays were performed by *Agrobacterium*-mediated vacuum infiltration [35]. Plants were vacuum-infiltrated two times within a 10-day interval.

### Real-time PCR to detect viral titers in challenged plants

Equal amounts of genomic DNA were used for analysis. Real-time PCR was performed with Power SYBR™ Green PCR Master mix (Applied Biosystems, Foster City, California, United States) and ran in ABI7900HT. Forward primer 5’-CTGCACAATGTGGGACCCTTTG-3’ and reverse primer 5’-CTTCGCCCTGATGACAGAGATC-3’ were used for the amplification of viral DNA-A of either ICMV-Dha (128–290 nt) or ICMV-SG (127–289 nt). All samples were run in triplicate and the data was analyzed with RQ manager at a preset threshold cycle value (Applied Biosystems, Foster City, California, United States). The *Jatropha curcas rbcL* DNA served as an internal control using forward primer 5’-GGAGTTCCGCCTGAGGAAG-3’ and reverse primer 5’-CTTCTCCAGCAACGGGCTC-3’. As described by Prisco *et al*. [36], the relative quantification of virus titers was determined based on the value of the Ct, using the comparative C_T_ method and the formula 2^−ΔΔCt^.

### Southern blotting

Genomic DNA was digested with restriction enzymes and separated on 0.8% agarose gels. The gels were processed and resolved DNA bands transferred to a nylon Hybond-N^+^ membrane (GE Biosciences, Buckinghamshire, United Kingdom) following standard procedures [13]. Membranes were hybridized with a CaMV 2 × 35S promoter probe which included the double enhancers with 3’ ends at −76 (A) of 35S promoter. The probes were DIG-dUTP-labeled by PCR using a PCR DIG probe synthesis kit (Roche Shanghai, China) according to the manufacturer’s instructions and signals were detected by autoradiography.

## Additional files

Additional file 1: Figure S1. Alignments for the fragments (A: fragment 1, B: fragment 2, C: fragment 3) between ICMV-Dha and relative strains. *indicated ICMV strains identified from *Jatropha*. Additional file 2:
**Sequence of the synthetic 35S promoter harboring a double enhancer used in this report.**

## Abbreviations

ACMV: African cassava mosaic virus
Bp: base pair
CpCDPKV: *Chickpea chlorotic dwarf Pakistan virus*
CP: Coat protein
dsRNA: ds, Double-stranded RNA
EACMCV: East African cassava mosaic Cameroon virus
EACMV: East African cassava mosaic virus
hpRNA: Hairpin RNA
hpRNAi: Hairpin RNA-mediated RNAi
ICMV-Dha: Indian cassava mosaic virus Dha strain
ICMV-SG: Indian cassava mosaic virus SG strain
JcMD: *Jatropha curcas* mosaic disease
GPMLCuV: Gossypium punctatum mild leaf curl virus.kb, kilobases
PCR: polymerase chain reaction
PDR: Pathogen-derived resistance
qPCR: quantitative polymerase chain reaction
Rep: Rep Replication associated protein
RNAi: RNA interference
SACMV: South African cassava mosaic virus
siRNA: Small interfering RNA
SLCMV: Sri Lankan cassava mosaic virus
TAIL-PCR: Thermal asymmetric interlaced PCR
TrAP: transcriptional activator protein
Ren: Replication enhancer protein
