# Supplementary material for: Engineering geminivirus resistance in Jatropha curcus
Source: Biotechnol Biofuels. 2014 Oct 21;7:149. doi: 10.1186/s13068-014-0149-z (PMC4210599; doi:10.1186/s13068-014-0149-z)
Supplement: Additional file 1: Figure S1. — Alignments for the fragments (A: fragment 1, B: fragment 2, C: fragment 3) between ICMV-Dha and relative strains. *indicated ICMV strains identified from Jatropha. [file 13068_2014_149_MOESM1_ESM.docx]

> Synthetic 35S promoter harbouring a double enhancer

Cgacactctcgtctactccaagaatatcaaagatacagtctcagaagaccaaagggctattgagacttttcaacaaagggtaatatcgggaaacctcctcggattccattgcccagctatctgtcacttcatcaaaaggacagtagaaaaggaaggtggcacctacaaatgccatcattgcgataaaggaaaggctatcgttcaagatgcctctgccgacagtggtcccaaagatggacccccacccacgaggagcatcgtggaaaaagaagacgttccaaccacgtcttcaaagcaagtggattgatgtgataacatggtggagcacgacactctcgtctactccaagaatatcaaagatacagtctcagaagaccaaagggctattgagacttttcaacaaagggtaatatcgggaaacctcctcggattccattgcccagctatctgtcacttcatcaaaaggacagtagaaaaggaaggtggcacctacaaatgccatcattgcgataaaggaaaggctatcgttcaagatgcctctgccgacagtggtcccaaagatggacccccacccacgaggagcatcgtggaaaaagaagacgttccaaccacgtcttcaaagcaagtggattgatgtgatatctccactgacgtaagggatgacgcacaatcccactatccttcgcaagaccttcctctatataaggaagttcatttcatttggagaggacacgctgaaatcaccagtctctctctacaaatctatctct
