# Supplementary figures and images for: Engineering geminivirus resistance in Jatropha curcus
Source: Biotechnol Biofuels. 2014 Oct 21;7:149. doi: 10.1186/s13068-014-0149-z (PMC4210599; doi:10.1186/s13068-014-0149-z)

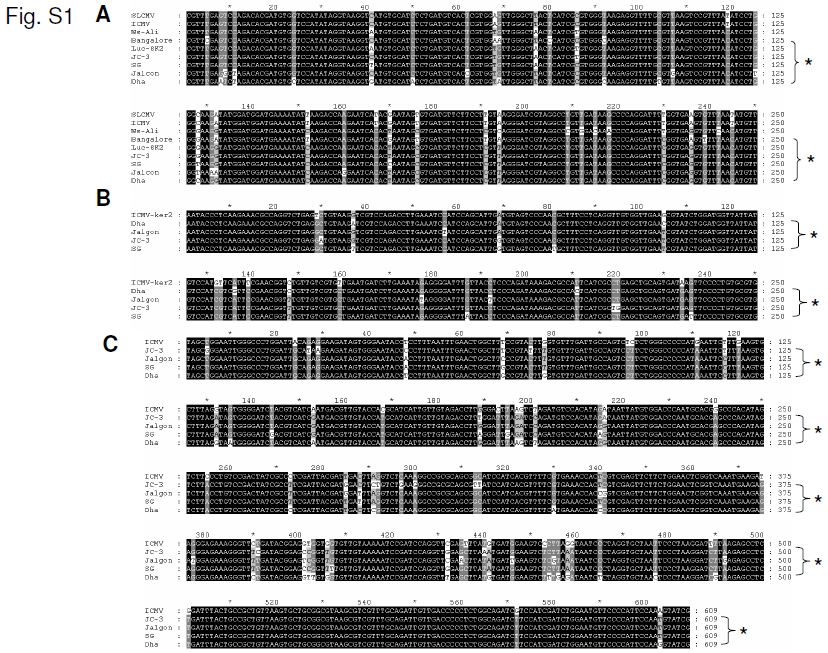

Supplement: Additional file 2: — Sequence of the synthetic 35S promoter harboring a double enhancer used in this report. [file 13068_2014_149_MOESM2_ESM.jpeg]
